# Inflammation and Proliferation Act Together to Mediate Intestinal Cell Fusion

**DOI:** 10.1371/journal.pone.0006530

**Published:** 2009-08-06

**Authors:** Paige S. Davies, Anne E. Powell, John R. Swain, Melissa H. Wong

**Affiliations:** 1 Department of Dermatology, Knight Cancer Institute, Oregon Stem Cell Center, Oregon Health & Science University, Portland, Oregon, United States of America; 2 Department of Cell and Developmental Biology, Oregon Health & Science University, Portland, Oregon, United States of America; Charité-Universitätsmedizin Berlin, Germany

## Abstract

Cell fusion between circulating bone marrow-derived cells (BMDCs) and non-hematopoietic cells is well documented in various tissues and has recently been suggested to occur in response to injury. Here we illustrate that inflammation within the intestine enhanced the level of BMDC fusion with intestinal progenitors. To identify important microenvironmental factors mediating intestinal epithelial cell fusion, we performed bone marrow transplantation into mouse models of inflammation and stimulated epithelial proliferation. Interestingly, in a non-injury model or in instances where inflammation was suppressed, an appreciable baseline level of fusion persisted. This suggests that additional mediators of cell fusion exist. A rigorous temporal analysis of early post-transplantation cellular dynamics revealed that GFP-expressing donor cells first trafficked to the intestine coincident with a striking increase in epithelial proliferation, advocating for a required fusogenic state of the host partner. Directly supporting this hypothesis, induction of augmented epithelial proliferation resulted in a significant increase in intestinal cell fusion. Here we report that intestinal inflammation and epithelial proliferation act together to promote cell fusion. While the physiologic impact of cell fusion is not yet known, the increased incidence in an inflammatory and proliferative microenvironment suggests a potential role for cell fusion in mediating the progression of intestinal inflammatory diseases and cancer.

## Introduction

Cell fusion between bone marrow-derived cells (BMDCs) and somatic cells has been reported in a number of different organ systems as an intriguing means for tissue regeneration in response to injury [1]–[10]. The low incidence described in early studies led critics to suggest that cell fusion was physiologically inconsequential. However, two groups recently published that chronic inflammation can potentiate this process in the brain, muscle, liver and heart [11], [12] suggesting that physiologic mediators can affect cell fusion. We have previously reported that BMDCs fuse with intestinal stem or progenitor cells after γ-IR-induced epithelial injury and that cell fusion is markedly increased in intestinal tumors [8]. Intestinal tumors are well-characterized by chronic inflammation [13]–[16] leading to the possibility that inflammation plays an important role in tumor progression. Notably, patients with chronic intestinal inflammation have a higher incidence for developing colorectal cancer [17], [18]. This highlights the importance of understanding how the microenvironment impacts cell fusion and if this process contributes to tumorigenesis.

## Results and Discussion

**Figure 1:**
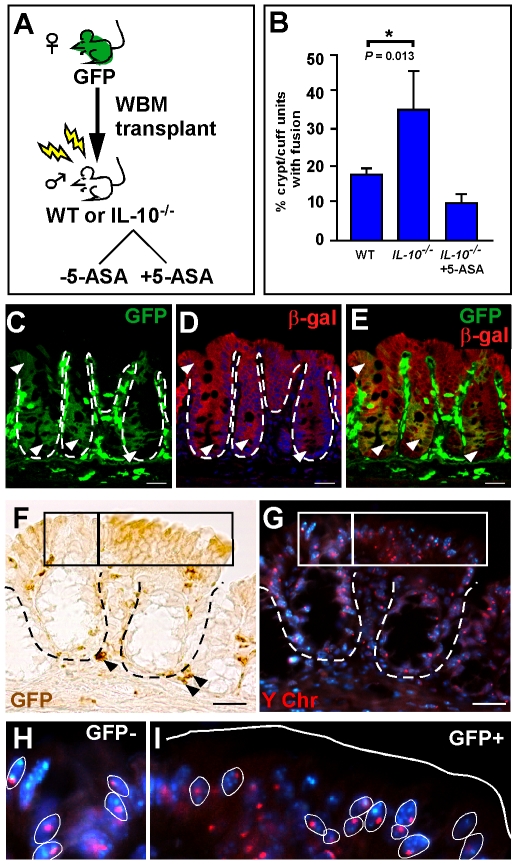
Inflammation promotes cell fusion between bone marrow-derived cells (BMDCs) and intestinal epithelium. (A) Schematic representation of experimental design. Whole bone marrow (WBM) from a female GFP-expressing donor mouse was transplanted into lethally irradiated wild-type (WT) or *IL-10^−/−^* male mice. A subset of *IL-10^−/−^* recipient mice were given the anti-inflammatory drug, 5-ASA. (B) Comparison of cell fusion in colonic epithelium between recipient mice. Cell fusion is quantified as the percentage of crypt/cuff units with at least one GFP-expressing cell. (C–E) Single plane confocal image of a colon cross-section from a ROSA mouse transplanted with GFP WBM. GFP expression (C, green) and β-gal expression (D, red) exist in the same cell (E, yellow) indicating fusion between the donor and recipient cell. Arrowheads denote fused epithelium on the cuff and in the crypts. (F–I) GFP-expressing epithelial cells in transplanted *IL-10^−/−^* colons are also fusion products, as determined by co-expression of GFP (F, brown, right box) and the Y chromosome (G, red, right box). (H & I) Higher magnification of GFP-negative and GFP-positive boxed regions from panels F & G. Y chromosome is found in Hoechst stained nuclei (blue, examples circled in white). Solid white line denotes epithelial/luminal border; dashed white lines indicate epithelial/mesenchymal border. Bars = 25 µm.

To identify if well-characterized tumor microenvironmental factors mediate intestinal cell fusion, we set out to directly test the hypothesis that cell fusion is enhanced by inflammation. Utilizing the established mouse model of colonic inflammation, the *IL-10^−/−^* mouse [19]–[21], we compared the incidence of epithelial cell fusion in mice transplanted with green fluorescent protein (GFP)-expressing whole bone marrow (WBM) with those treated with the anti-inflammatory drug, 5-aminosalicylic acid (5-ASA), or to wild-type (WT) transplanted mice (Figure 1A). Analyses of peripheral blood after WBM transplantation revealed high levels of donor-blood reconstitution in all analyzed mice (>90% GFP expression, data not shown). Cell fusion between donor BMDCs and the colonic epithelium was identified by co-expression of both the donor marker, GFP, and the WT epithelial marker, β-galactosidase (β-gal) by confocal microscopy (Figure 1C–E). GFP epithelial expression was detected by immunohistochemical analysis using antibodies to GFP or by direct fluorescence (Figure S1A–F). Proper controls were analyzed to confirm that epithelial GFP-expression was not due to artifact (Figure S2). GFP-expressing cells residing in the epithelial compartment were confirmed to be predominantly epithelial cells based upon morphology and co-expression of E-cadherin (Figure S1G–J). Phenotypically distinct CD45-positive cells (intra-epithelial lymphocytes) were also present in this compartment, but were much smaller and did not extend to the apical border (Figure S1K–O). Together, these rigorous standards definitively establish that GFP-expressing epithelial cells of both the small and large intestine can be accurately identified.

Cell fusion was analyzed in the chronically inflamed colon from male *IL-10^−/−^* mice that were transplanted with GFP-expressing WBM from a female donor. Detection of the recipient marker (Y-chromosome) by *in situ* hybridization and the donor marker (GFP) by immunohistochemical analysis provides an additional approach to analyze cell fusion (Figure 1F-I). The presence of co-localized Y-chromosome in GFP-expressing cell regions (Figure 1I) indicates that cell fusion occured in the presence of chronic inflammation. Controls verifying the specificity of the Y-chromosome probe are presented in Figure S3.

We chose to use epithelial GFP-expression as the basis for quantifying cell fusion based upon two criteria. First, cell fusion was initially confirmed in all experimental groups and in all recipient backgrounds used in the studies reported here. This was established using confocal microscopy and immunohistochemical, or histochemical co-detection of donor and recipient markers in the same epithelial cell (Figure 1C-I and Figure S4A–C). Second, we and others have reported that in all of the mice surveyed for cell fusion, the donor marker predominatly expresses the recipient marker [1], [7], [8], [11], [12], [22], or in other words, presence of the donor marker in the intestinal epithelium does not support transdifferentiation, a change in cell fates from the BMDC to a non-hematopoietic cell type. Based upon these criteria, cell fusion was quantified in each animal by counting the percentage of crypt units (crypt/villus or crypt/cuff in the small intestine or colon, respectively) that contained GFP-expressing epithelial cells in a total of 1500 crypt units.

Interestingly, we observed a dramatically higher amount of epithelial cell fusion in WBM-transplanted WT mice than what we had previously reported [8]. This observation is the result of optimization of our transplantation protocols for intestinal cell fusion, including the use of a more robust and detectable GFP-expressing transgenic line for donor bone marrow (Osb-Y01) [23], [24], more effective GFP detection by antibody staining, and establishing stringent quantification methods. We now report that fusion within the intestinal epithelium is detected at a level of 37.3±3.6% in the distal small intestine (DSI; *n* = 10, Figure S4D) and 20.6±2.1% (*n* = 4) in the colon. The prominent level of cell fusion sets the intestine apart from other systems where only low levels are observed [1]–[7], [9], [10], suggesting that there is a physiologically important role for cell fusion in self-renewing tissues.

In assessing the role of inflammation, direct comparison of cell fusion in colons from *IL-10^−/−^* mice (chronic inflammation) with WT controls revealed a significant increase (*IL-10^−/−^*: 35.2±9.6%, *n* = 3; WT: 19.04±1.1%, *n* = 9; *P* = 0.013) (Figure 1B). To further implicate the presence of local intestinal inflammation in promoting cell fusion, we treated *IL-10^−/−^* mice with the anti-inflammatory drug, 5-ASA [25], a standard therapy for inflammatory bowel disease in humans. Treatment with 5-ASA resulted in a marked decrease in cell fusion (8.5±2.7%, *n* = 3; Figure 1B) compared to untreated *IL-10^−/−^* mice. This dramatic effect of modulating microenvironmental inflammation on cell fusion is depicted in tissue sections from each of the experimental groups and presented in Figure S5. We confirmed by quantitative reverse transcriptase-polymerase chain reaction (qRT-PCR) that *IL-10^−/−^* intestines had heightened inflammation and that treatment with 5-ASA greatly suppressed the inflammatory response [26], [27]; (Mchr1, Melanin-concentrating hormone receptor1 and IL-1β, Interleukin-1β, Table S1).

**Figure 2:**
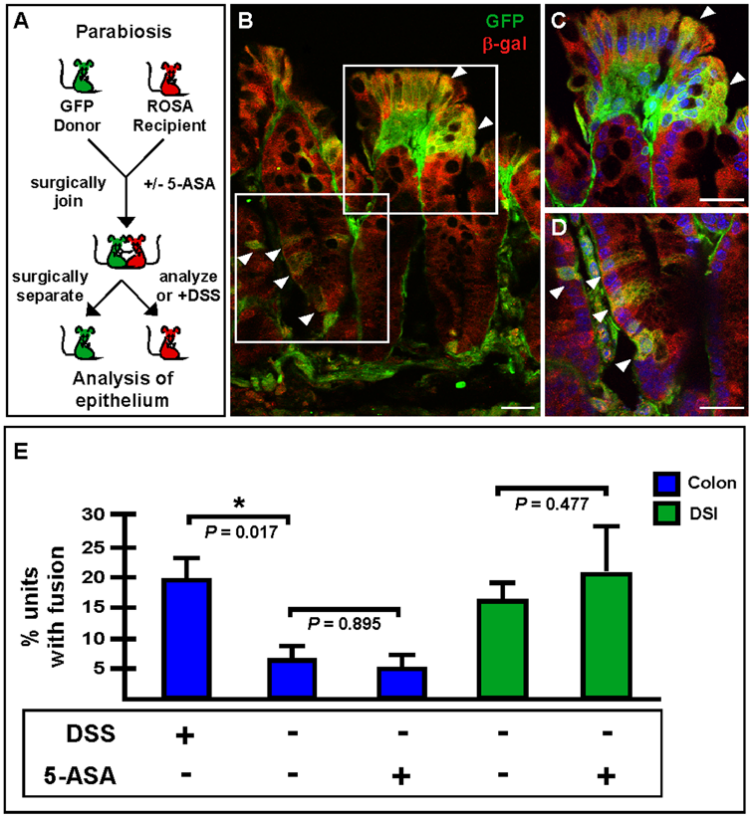
Intestinal cell fusion persists at low levels in a non-damage model system. (A) Schematic representation of parabiosis experimental design. GFP and ROSA mice were surgically joined. (B–E) Extensive cell fusion was observed in colons from DSS-treated animals. (B) Single plane confocal microscopy images of GFP (green) and β-galactosidase (red) detected by antibodies demonstrate fusion by co-localization in yellow. Arrowheads denote fused cells. (C & D) depict higher magnifications of the boxed regions in panel B. Nuclei were visualized with the Hoechst dye (blue). Bars = 25 µm. (E) Cell fusion in DSS-treated animals was significantly increased over non-treated animals (*P* = 0.017). When the animals were given 5-ASA during parabiosis to inhibit inflammation, there was no difference in fusion levels in colon (blue bars; *P* = 0.895) or DSI (green bars; *P* = 0.477), however, a baseline level of fusion existed in both tissues.

It is well-established that γ-IR also induces an inflammatory response in the intestine [28]. Given that the transplantation procedure involved γ-IR, we utilized a parabiosis approach to introduce traceable bone marrow without γ-IR. Surgically joined parabiotic mouse pairs were maintained together for 4–6 weeks to establish a shared circulating blood supply [29]–[31], which was confirmed by flow cytometry (data not shown). After the mice were separated, intestinal inflammation was induced by administration of dextran sodium sulfate (DSS), a well-documented protocol for eliciting inflammation in the mouse intestine and colon [32] (Figure 2A). The DSS phenotype can be appreciated on both gross morphologic and cellular levels (WT compared to DSS-treated, Figure S6). Again, cell fusion was apparent in the DSS-induced colons of these animals by co-detection of donor and recipient markers using confocal microscopy (β-gal and GFP; Figure 2B–D). Distinct epithelial regions expressing both GFP and β-gal were readily detectible in both the crypt cuff (Figure 2B,C; arrowheads) and in the colonic crypt (Figure 2 B,D; arrowheads). Quantification of cell fusion revealed a statistically significant increase in the DSS-treated parabiotic partners compared to untreated controls (Figure 2E; WT: 5.8±3.4%, *n* = 5; DSS-treated: 19.6±2.6%, *n* = 4; *P* = 0.017). These data, along with our observations in the WBM-transplanted mice strongly implicate inflammation as a key mediator for pathologically-induced cell fusion in the intestine.

In support of a physiologic role for intestinal epithelial cell fusion, an appreciable baseline level of cell fusion was observed in non-DSS treated parabiotic pairs in both the colon (-DSS, 5.8±3.4%, *n* = 5) and DSI (-DSS, 15.0±3.2%, *n* = 5) (Figure 2E). Even though parabiosis surgery is well-accepted as a “non-damage” model, there is considerable post-surgery stress to the animal resulting in weight loss, and it is possible that intestinal injury occurs during or immediately after the surgical procedure. To rule out the possibility of surgically-induced inflammation that could potentially create an artificial baseline level of cell fusion within the intestinal epithelium, we repeated the parabiotic experiment by joining GFP and ROSA mice along with oral administration of an anti-inflammatory drug cocktail during and after the surgery (Figure 2A). In these animals, the baseline level of cell fusion persisted and was unchanged relative to the untreated animals in both the colon (5-ASA treated: 5.3±2.0%, *n* = 5, *P* = 0.895) and DSI (5-ASA treated: 21.3±7.8%, *n* = 5, *P* = 0.477) (Figure 2E). Further, we confirmed by qRT-PCR that animals receiving an anti-inflammatory drug regimen had minimal epithelial inflammation (Table S1). This is in agreement with the data presented in Figure 1B, which showed *IL-10^−/−^* mice treated with 5-ASA after transplantation also displayed appreciable levels of cell fusion. Together, these observations highlight the existence of an endogenous baseline level of epithelial cell fusion in the intestine, suggesting that the nature of rapidly self-renewing epithelium may sensitize or prime it for fusion with circulating BMDCs under certain microenvironmental conditions. These findings strongly suggest that additional factors are important for the fusion process in the intestine.

Currently, reports in other organ systems show that baseline levels of cell fusion are relatively non-detectable [11], [12], [33]. Important differences between these other organ systems and the intestine is that the intestinal epithelium is a rapidly renewing, highly proliferative tissue that dynamically responds to its microenvironment. An additional distinction between the intestine and the other somatic organs lies in the host fusogenic cell. We have previously reported that BMDC fusion occurs with a stem or progenitor population in the intestine [8], whereas in other tissues fusion takes place with differentiated cells [1], [7], [12], [22]. These differences along with the respective disparity in homeostatic cell fusion levels suggest that host-cell proliferative status may be a factor in the fusion process. It is well established that γ-IR elicits intestinal microenvironmental inflammation [28], and that the epithelium undergoes massive apoptosis that peaks within the first 24 h post-irradiation [34] accompanied by a proliferative response [35]. Further, we have previously shown that γ-IR also stimulates the Wnt signaling pathway, a critical regulator of intestinal epithelial proliferation [36]. Taken along with our observation that fusion is increased in a tumor setting [8], these elements implicate cell death or proliferation signals as possible additional factors that promote cell fusion.

**Figure 3:**
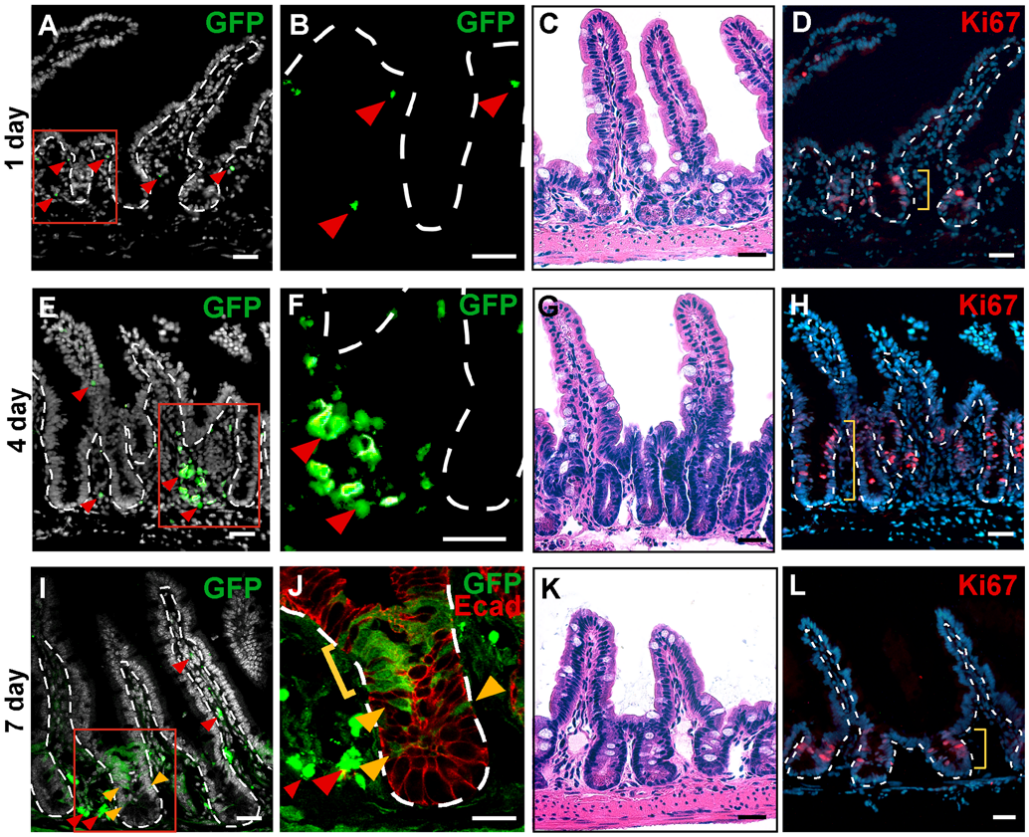
Increased epithelial proliferation occurred after gamma-irradiation. Wild-type (WT) mice were transplanted with GFP-expressing whole bone marrow (WBM). The distal small intestine was analyzed at 24 h increments for 1 week. (A–B) At 1 day post-transplantation, few GFP-positive cells (green) were located in the mesenchyme (arrowheads) and none were found in the epithelium. (C–D) H&E and Ki67 detection (red) with Hoechst dye (blue) indicated normal morphology at one day post-transplant. (E–F) At 4 days post-transplant, more GFP-positive cells were found surrounding the crypt (arrowheads), while none were detected in the epithelium. (G–H) H&E and Ki67 staining (red) revealed a dramatic increase in proliferation of the crypts. (I–J) By 7 days post-transplant, single plane confocal microscopy depicts the presence of GFP-positive cells in the mesenchyme surrounding the crypt as well as in the villi core (red arrowheads). GFP-positive epithelium was observed in the stem cell (yellow arrowheads) and transient-amplifying (yellow bracket) zones of crypts. Epithelial cells are marked with antibodies against E-cadherin (red). (K–L) H&E and Ki67 staining depicted morphology close to normal by 7 days post-transplant. Dashed white lines indicate epithelial/mesenchymal border. Red boxes in (A,E,I) are displayed in higher magnification in (B,F,J). Yellow brackets denote the depth of the Ki67-positive cells in (C,G,K). The nuclear dye Hoechst is depicted in grayscale in (A,E,I) and in blues in (D,H,L). D & H are the same tissue sections as A & E, respectively. Bars = 25 µm.

To gain additional insights from the pre-fusion intestinal microenvironment, we detailed the temporal events surrounding the generation of cell fusion hybrids. The dynamic trafficking of GFP-expressing BMDCs to the intestine was defined at various early time points post-transplantation. Since our initial observations implicated progenitor cells as the host fusion partner [8], we focused our analyses on the stem cell niche. At 1 day post-transplant, the first detectable GFP-positive BMDCs were present scattered around the crypt region in the intestinal mesenchymal compartment (Figure 3A, B; arrowheads). By 4 days post-transplant, an appreciable level of GFP-expressing BMDCs populated the intestine, but GFP-expressing epithelium was not yet observed (Figure 3E, F; arrowheads). Cell fusion in the epithelial compartment (Figure 3J; yellow brackets and arrowheads) was routinely detected 7 days post-transplant and was accompanied by high levels of GFP-expressing cells in the mesenchyme (Figure 3I,J; red arrowheads). The arrival of GFP-expressing BMDCs into the intestine coincided with a striking increase in proliferation of the intestinal epithelium, appreciated both histologically by Hematoxylin and Eosin (H&E) staining and by Ki67 antibody staining (Figure 3C,D,G,H,K,L; yellow brackets). Intriguingly, the dramatic proliferative epithelial response coincident with clustering of GFP-expressing BMDCs in the stem cell niche suggested that intestinal cell fusion may also be governed by the proliferative status of the recipient cell.

The homeostatic, or baseline, levels of cell fusion observed in both transplanted mice with suppressed inflammation and parabiotic mice may be due to the intrinsic proliferative nature of the intestinal crypt. Approximately 60% of crypt cells are actively engaged in the cell cycle [35], [37] supporting rapid self-renewal of the epithelium. We and others [35] have shown that this proliferative zone is expanded in response to γ-IR (Figure 3G,H). However, in other tissues where cell fusion occurs after γ-IR, it is reported that the host fusion partner is a differentiated cell type that is not known to be actively cycling [1], [11], [22]. Despite this, notexin-induced injury in skeletal muscle, where BMDC fusion has been described, results in a transient increase in cell numbers [38], suggesting that these differentiated cells might be capable of entering the cell cycle. Based upon this observation and our data in the intestine, we hypothesized that host cell proliferative status is important in driving cell fusion. Therefore, to determine if entry into the cell cycle might also be stimulated in a subset of other organs after γ-IR, we surveyed for cycling cells in the liver and skeletal muscle using antibodies to Ki67. A visible increase of Ki67-positive cells was observed (Figure S7), further supporting the idea that cell cycle status of the host cell within these organs may also mediate cell fusion.

**Figure 4:**
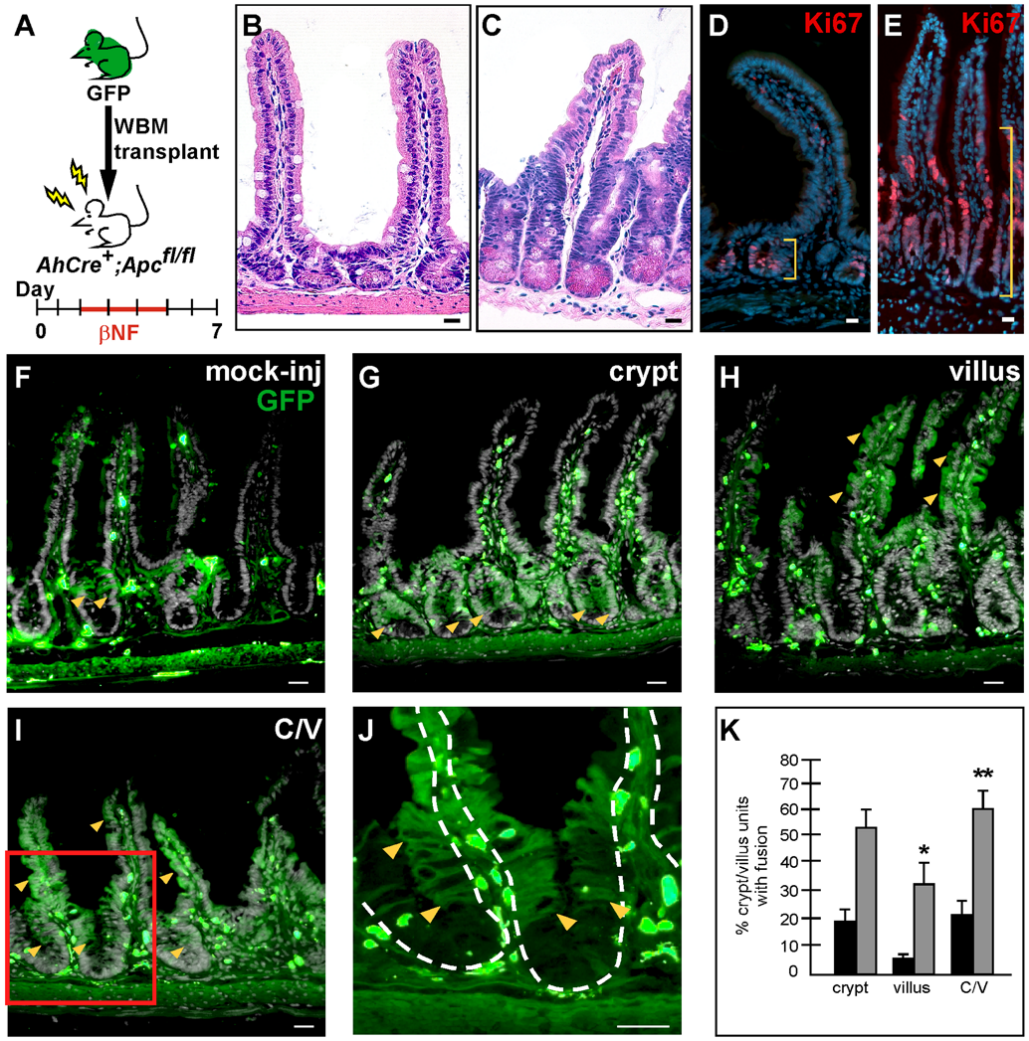
Increased epithelial proliferation correlates with increased cell fusion. (A) Schematic representation of experimental design. *AhCre^+^;Apc^fl/fl^* mice were transplanted with GFP-expressing whole bone marrow (WBM) on day 0. Two days later, Cre recombinase was induced by β-naphthoflavone (β-NF) administration for 4 consecutive days (days 2–5). Mice were sacrificed on day 7 and the distal small intestine analyzed for fusion (B–E). The intestinal-specific deletion of *Apc* resulted in an extensive hyperproliferation of crypt cells compared to wild-type (WT) mice, as seen by H&E stain (C vs. B) and Ki67 staining (red, indicated by yellow brackets; E vs. D). (F–K) Detection of GFP-expressing cells (green; yellow arrowheads mark examples) denoting cell fusion was increased in the *AhCre^+^;Apc^−/−^* mice compared to mock-injected WT mice. Three patterns of cell fusion were observed: (G) crypt-only, (H) villus-only, (I) both crypt and villus regions in one crypt/villus unit. Panel (J) is a higher magnification of the red box in panel (I) demonstrating that the Paneth cell region at the base of the crypt remained GFP-negative. Solid white lines denote epithelial/luminal border; dashed white lines indicate epithelial/mesenchymal border. Bars = 25 µm. (K) A significant increase in fusion was observed in villus only (*P* = 0.011) and crypt/villus (*P* = 0.009) *AhCre^+^;Apc^−/−^* mice (gray bars) compared to mock-injected WT mice (black bars).

Therefore, to directly implicate epithelial proliferative status as a contributing host factor in promoting cell fusion, we utilized a mouse model in which we could temporally manipulate epithelial proliferation in the intestine. The previously described intestinal-specific, inducible *AhCre* mouse [39] harboring floxed *Apc* alleles [40], results in a dramatic induction of intestinal epithelial proliferation upon Cre activation [41] and Figure 4C,E. We showed by H&E that an increase in immune infiltrate was not readily apparent during the timeframe in which epithelial proliferation was stimulated (compare Figure 4B,C). Further, qRT-PCR showed that there was not an increase in inflammation in these mice (Table S1). To evaluate cell fusion in this proliferative model, we transplanted *AhCre^+^;Apc^fl/fl^* mice on day 0, induced epithelial proliferation on day 2, then analyzed cell fusion in the intestine on day 7 (Figure 4A). Dramatically, a significant increase in epithelial cell fusion within the crypt and villus, compared to mock-injected controls, was observed (Figure 4F–K). Cell fusion in crypt/villus units displayed three distinct patterns: fusion restricted to the crypts, fusion on the villus only, and fusion in both crypt and villus epithelium (Figure 4G–J). Differences in each of these three patterns were significant when compared to mock-injected control intestines (crypt: *P* = 0.056; villus: *P* = 0.011; crypt/villus: *P* = 0.009; mock injected *n* = 5, *AhCre^+^;Apc^−/−^ n* = 7). Because the induction of proliferation occurs over a window of 4 days, the differences in the crypt, villus or crypt/villus fusion expression patterns likely represented different kinetics of cell fusion and subsequent expansion of progeny. For example, it is possible that fusion in the crypt epithelium represents an initial fusion event in a proliferative cell that occurred only a short time before analysis (perhaps on day 5–6). Likewise, GFP-expressing epithelia in both the crypt and villus might represent an early fusion event in a crypt-based progenitor cell, perhaps on day 2. Notably, crypt-based differentiated Paneth cells which have a >20 day turnover [42], remain unmarked and are not descendents from the cell fusion event (Figure 4J).

Importantly, detection of cell fusion only on the villus where proliferative cells do not normally reside, strongly implicated the proliferative status of the host cell as a critical component of cell fusion. Noticeably, each crypt/villus unit had extensive GFP-expressing cells which could argue for a more rapid expansion of progeny from the original fusion event. However, the fact that there were significantly more total crypt/villus units harboring at least one GFP-expressing cell indicated there were also more initial cell fusion events (*P* = 0.009). Our assay cannot distinguish between whether the host fusion target is a progenitor or if it is a cell actively engaged in the cell cycle. Regardless, our data indicates that the host cell must be receptive or primed for the fusion process. Importantly, these observations suggest that proliferative capacity of the host cell contributes to promote cell fusion in the intestinal epithelium.

**Figure 5:**
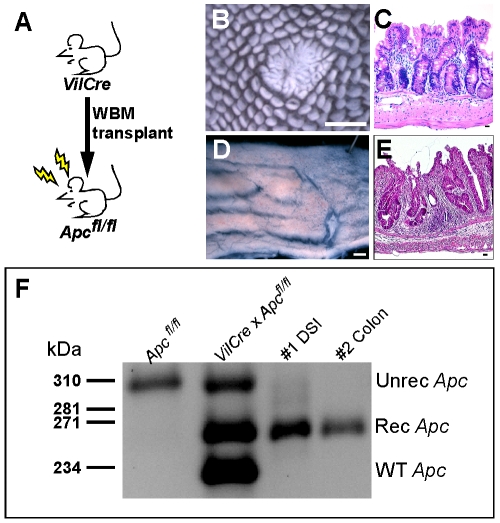
Bone-marrow/epithelial cell fusion causes genetic reprogramming. (A) Schematic diagram of transplantation scheme. Whole bone marrow (WBM) from mice expressing Cre recombinase driven by the intestinal epithelial-specific *Villin* promoter (VilCre) was transplanted into recipient mice that were homozygous for floxed *Apc*. Resulting intestinal phenotypes were observed in transplanted mouse intestine by wholemount analysis as polyps (B) in the distal small intestine (DSI) and as thickened unorganized epithelia (D) in the colon. H&E staining confirmed the phenotypic morphology (C,E). Bars in B & D = 1 mm, bars in C & E = 25 µm. (F) To confirm that the phenotype was the result of recombination at the *Apc* allele, PCR analysis of epithelium from recipient mice using primers that specifically detect the recombined *Apc* allele was performed. The 258 bp band was present in the transplanted DSI and colon samples, indicating cell fusion by activation of Cre-recombinase.

Perhaps the most pressing question relating to *in vivo* cell fusion is if the generated cell fusion hybrids have a physiologic impact on normal organ function. Although it is apparent that these intestinal cell fusion hybrids retain an overt epithelial phenotype, it is unclear if the BMDC transcriptome is modified. To explore the possibility that cell fusion results in nuclear reprogramming of the donor genome, we transplanted WBM from mice harboring a *Villin-Cre* transgene [43] into recipient mice homozygous for the floxed *Apc* allele [40](Figure 5A). Villin is an epithelial-specific promoter and Cre recombinase is not expressed in any of the blood lineages under this context [43]. Therefore, functional Cre recombination of the *Apc* allele would only occur if Cre recombinase were activated, such as in the event of cell fusion between the BMDC (*Villin-Cre*) and the epithelial cell. The intestines from transplanted mice possessed hyperproliferative epithelial regions in both the distal small intestine and colon appreciated by wholemount analysis (Figure 5B,D) and morphologically by H&E (Figure 5C,E). The polyp-like region in the distal small intestine was reminiscent of Min mouse polyps [44] where the *Apc* allele is mutated. To confirm the phenotype was due to Cre mediated recombination of the *Apc* allele, we isolated DNA from intestinal tissue sections and performed PCR with primers specific to the recombined floxed *Apc* allele. In both the DSI and the colon, a 258 bp amplicon was identified, confirming that Cre recombinase had been activated within the tissue. This observation not only strongly supports the occurrence of cell fusion, but it importantly illustrated that these cell fusion hybrids can reprogram BMDC gene expression by activating an epithelial-specific promoter. While this functional evidence supports the implication that cell fusion can create a genetically distinct hybrid cell, the extent of reprogramming of the genome remains an intriguing and important future focus of investigation.

It is clear, from our studies presented here and previous reports [8], [11], [12], [22] that cell fusion between BMDCs and non-hematopoeitic tissues presents an important physiologic occurrence. Here, we report considerable baseline levels of cell fusion in the intestine under homeostatic conditions, greater than that reported in other organ systems [1], [11], [22]. Further, we demonstrate that intestinal cell fusion with BMDCs is mediated by both inflammation and cellular proliferation. A possible physiologic role for intestinal cell fusion may be to facilitate rapid regeneration of the epithelial barrier after injury. Because the intestinal epithelium is the largest surface barrier to the external environment, barrier maintenace is critical for the organism's survival. If cell fusion participates in this rapid response, the intestine is certainly poised to solicit fusion with both its intrinsic immune capacity and functional proliferation. While previous reports dismiss the importance of cell fusion or tie its potential to therapeutic gene replacement strategies, our data implicates cell fusion in a role to potentially impact inflammatory disease pathogenesis, including inflammatory bowel disease and cancer. Only by understanding the long-term fate of the epithelial cell fusion hybrid will we uncover its physiologic potential in both homeostasis and disease.

## Materials and Methods

### Ethics Statement

Mice were housed in a specific pathogen-free environment under strictly controlled light cycle conditions, fed a standard rodent Lab Chow (#5001 PMI Nutrition International), and provided water ad libitum. All procedures were approved and performed in accordance with the Oregon Heatlth and Science University animal ethics committee: the Oregon Health & Science University Institutional Animal Care and Use Committee. There are no human subjects involved in this study.

### Mice

The C57Bl/6, 129/Sv or ROSA [45] (WT), *IL-10^−/−^* [19], [20] and *Villin-Cre* [43] mice were obtained from The Jackson Laboratory. *AhCre* mice[39] were kindly provided by Dr. Douglas Winton (University of Cambridge). Osb-Y01 (GFP) [23], [24] and *Apc^580S^* mice (designated as *Apc^fl/fl^* in the unrecombined state and *Apc^−/−^* after recombination) [40] were bred in-house.

### Bone Marrow Transplantation

Whole bone marrow (WBM) transplantation was carried out as we have previously described with some modifications [8]. Briefly, 6-week-old recipient male WT, *IL-10^−/−^*, *Apc^fl/fl^*, or *AhCre^+^*;*Apc^fl/fl^* mice received whole-body γ-IR (12 Gy: in two 6 Gy doses, 4 hours apart). BMDCs were harvested from 5- to 12-week-old donor GFP [23], [24] or *Villin-Cre* [43] mice using standard procedures [46], filtered to obtain a single-cell suspension and resuspended in Hank's balanced salt solution supplemented with 3% fetal bovine serum and 10 mM HEPES. A total of 1×10^7^ WBM cells were then injected retro-orbitally into recipient mice. To confirm hematopoietic engraftment, peripheral blood leukocytes were isolated from recipient mice as previously reported [47] and analyzed using a Becton Dickinson FACSCalibur.

### Parabiosis

Parabiosis surgery was performed between GFP and ROSA mice (*n* = 5 pair for WT, *n* = 5 pair for 5-ASA, *n* = 4 pair for DSS treatments) as described previously [47]. Briefly, pairs of 6- to 12-week age- and weight-matched mice were surgically joined from the elbow to knee. Each parabiotic partner was given recombinant human granulocyte colony-stimulating factor (250 µg/kg subcutaneously; Amgen) for 4 days starting at day 17 post-surgery [29]. Mice were separated approximately 7 weeks after surgery and intestinal tissue analyzed.

### Manipulation of intestinal inflammation

To suppress inflammation in *IL-10^−/−^* mice, 5-aminosalicylic acid (5-ASA) was administered in the drinking water at the time of WBM transplantation (500 ppm 5-ASA/5mM Sodium Phosphate; Sigma). Mice were analyzed 3–7 months later. For parabiosis studies, animals were administered 5-ASA 1 week prior to surgery and continually until surgical separation. Meloxicam (a Cox-2 inhibitor; Boehringer Ingelheim) was concurrently administered for 4 days post-surgery. To induce inflammation in parabiotic mice, dextran sodium sulfate (DSS; TdB Consultancy AB) was given in drinking water (2.5% DSS in 5% sucrose) [32] 1 week after separation followed by regular water for 1 week, at which point the animals were sacrificed and analyzed.

### Intestinal proliferative model

To examine enhanced proliferation in the mouse intestine, we crossed *Apc^fl/fl^* [40] mice to the AhCre intestinal-specific inducible mouse line [39]. *AhCre^+^; Apc^fl/fl^* progeny were induced by intraperitoneal injection of β-naphthoflavone (β-NF; Sigma) dissolved in corn oil (80 mg/kg) for four days [41] and analyzed 2 days later. For transplantation studies, β-NF injections were initiated two days post-transplant.

### Intestinal analysis of transplanted and parabiotic mice

Cell fusion was confirmed by co-localization of GFP expression developed for brightfield and Y-chromosome fluorescence *in situ* hybridization, or for fluorescent detection by confocal microscopy with co-staining of antibodies for β-gal (1∶500, Immunology Consultants Laboratory, Inc.) and GFP as reported previously [8] (*n* = 18).

Mice were analyzed at varying times post-transplantation (*IL-10^−/−^* studies: 3 and 7 months post-transplant, *n* = 7; proliferation studies: *n* = 13; WT transplants: 1–11 months for colon, *n* = 9, 3–11 months for DSI, *n* = 10; genetic recombination studies: 2–5 months, *n* = 11). Analysis of parabiotic pairs took place at time of separation (4–9 weeks; *n* = 10) or 3 weeks after separation for DSS studies (*n* = 4). Small intestine and colon was isolated *en bloc,* processed for wholemount imaging and subsequent frozen block preparation and sectioned as previously described [48]. Tissue sections (5 µm) were analyzed for GFP-expressing cells by using polyclonal antibodies to GFP (1∶500; Molecular Probes) and fluorescent secondary antibodies (1∶500, Alexa 488, Molecular Probes; 1∶500, Cy3 and 1∶250, Cy5, Jackson Immuno Research) or for brightfield detection by using biotin–avidin secondary antibodies and visualization with 3-3′-diaminobenzidine (DAB) according to the manufacturer's guidelines (Vector Laboratories). For controls, tissues were stained with anti-CD45 (1∶500; eBioscience), anti-E-cadherin (1∶1000; Zymed), and anti-laminin (1∶1000; Chemicon) followed by detection with appropriate fluorescent secondary antibodies. In some cases, tissue sections were also labeled with antibodies to the proliferation marker Ki67 (1∶500; Abcam). Nuclei were counterstained with Hoechst (33258; Sigma; 0.1 µg/ml). For H&E images, paraffin sections were prepared as previously described [48]. Sections were examined with a Leica DMR microscope and digital images were captured with a DC500 digital camera and IM50 Image Manager Software (Leica Microsystems) or confocal images were acquired using an IX81 Inverted Microscope equipped with Fluoview FV1000-Spinning Disc Confocal (Olympus) scan head and FV10 ASW 1.7 software (Olympus). Cy3 images were captured as grayscale and digitally converted to red images with Adobe Photoshop CS2 (Adobe Systems Inc.). In some instances, Hoechst or laminin images were converted to grayscale.

To examine the temporal dynamics of peripheral blood infiltration and fusion in the intestine, WBM-transplanted WT mice were analyzed 1–7 days post-transplantation (*n* = 2–6 for each time point). GFP and Ki67 expression was surveyed in the DSI by co-staining with antibodies as described above. H&E images were captured from paraffin tissues prepared from γ-IR treated mice at the same time points.

### Analysis for recombination of the *Apc* allele

DNA was isolated from 10 µm thick paraffin tissue sections from *Apc^fl/fl^* mice that had received WBM from a *Villin-Cre* donor. PCR for the recombined *Apc* allele was performed as previously reported [40]. The resulting bands mark various *Apc* status: Unrecombined = 314 bp, Recombined = 258 bp, Wildtype = 226 bp. Controls were run with the following primers (P3, P4, P5 from [40]): 5′GTTCTGTATCATGGAAAGATAGGTGGTC3′; 5′CACTCAAAACGCTTTTGA GGGTTGATTC3′; 5′GAGTACGGGGTCTCTGTCTCAGTGAA3′. Touchdown from 65°C to 55°C, followed by 14 cycles at 55°C. Experimental samples were run with a nested PCR as follows: 1^st^ PCR reaction: F-5′TAACCTGTTCTGCAGTATGTTATCATTC3′ R-5′GAGCACCCAGTACGCTTCTAGAG3′. Touchdown from 65°C to 52°C, followed by 9 cycles at 52°C; extension time of 4 minutes. 2^nd^ PCR reaction (P3 and P5 primers from [40]: F-5′GTTCTGTATCATGGAAAGATAGGTGGTC3′ R-5′GAGTACGGGGTCTC TGTCTCAGTGAA3′. Touchdown from 65°C to 55°C followed by 14 cycles at 55°C.

### Analysis of liver and skeletal muscle

WT mice were exposed to a single dose of whole-body γ-IR (9Gy) [11] and sacrificed 1–7 days later. Liver and skeletal muscle (quadicep, tibialis anterior and soleus muscles) were isolated and fixed in 4% paraformaldehyde and prepared as a frozen block. Tissue sections (10 µm) were co-stained for the proliferation marker Ki67 as described above, along with cell-type specific antibodies. Skeletal muscle was pretreated to eliminate auto-fluorescence by incubating tissue in sodium tetraborohydrate (10 mg/ml; Sigma), followed by subsequent staining for myosin heavy chain (Anti-myosin MY-32; 1∶750; Sigma) using a mouse-on-mouse detection kit (M.O.M.; Vector Labs) followed by secondary detection with Anti-Biotin Cy5 Streptavidin (1∶200; Jackson ImmunoResearch). Liver sections were initially stained with Ki67, imaged, and sequentially stained using rabbit anti-FAH (1∶10,000; a kind gift from Markus Grompe [49]) followed by Cy5 secondary detection. Nuclei were counterstained with Hoechst dye. Digital images were captured as described above. Ki67-positive cells were quantified from 7 distinct 20x fields of view containing approximately 3500 hepatocytes or for skeletal muscle, 4 distinct 40x fields of view containing approximately 500 nuclei.

### Inflammation assay

Quantitative reverse transcriptase polymerase chain reaction (qRT-PCR) was used to measure changes in the mRNA levels of Interleukin-1β (IL-1β) and Melanin-concentrating hormone receptor 1 [27] (Mchr1) in isolated epithelial and mesenchymal cell populations from transplanted mice. Epithelial cell populations were isolated using a modified Weiser preparation [40], [41] as we previously described [36]. Following epithelial cell isolation, mesenchymal cells were isolated by scraping the remaining tissue on a tissue sieve (Bellco Glass, Inc.) to dislodge the mesenchymal population. Total RNA was purified from each cell population and cDNA was synthesized as we have previously described [50]. qRT-PCR was performed using a SYBR Green-based assay and a 7900 HT Sequence Detector according to established protocols [29], [42], [43]. Each cDNA sample was analyzed in triplicate, along with triplicate samples of the endogenous reference gene, Glyceraldehyde-3-phosphate dehydrogenase (Gapdh). Primers used are listed as follows: Gapdh: F- 5′AAATATGACAACTCACTCAAGATTGTCA3′, R- 5′CCCTTCCACAATGCCAAAGT3′; Mchr1: F-5′GGTAATGGTGTCTGGCACTTTG3′, R-5′ GCCATAGCAGTCAGGAT GTAGGT3′; IL-1β: F-5′CGTGCTGTCGGACCCATATG3′, R-5′GCCCAAGGCCA CAGGTATTTT3′.

### Statistics

Cell fusion was quantified by reporting the total number of crypt/villus (DSI) or crypt/cuff (colon) units harboring at least one or more GFP-positive cell(s). A unit is defined as one villus and its adjacent crypt (DSI) or a single colonic crypt and its adjacent epithelial cuff (colon). For each animal, tissue sections at least 125 µm apart were quantified and at least 1500 units were examined. This quantification standard reports the percentage of units containing at least one fusion event. We do not quantitate on a per cell basis because this would overestimate the extent of cell fusion due to proliferative expansion of the initial fusion event. Statistical significance between experimental populations was determined using a Student's two-tailed, paired *t*-test or unpaired *t*-test as determined appropriate for each experimental scenario. *P* values <0.05 were considered statistically significant. Statistical analysis was performed using GraphPad Prism for Windows (GraphPad Software). All data are presented as the mean±s.e.m.

## Supporting Information

Figure S1 GFP-expressing cell type. Wild-type (WT) mice were transplanted with GFP-expressing whole bone marrow (WBM). (A–F) Five µm sections from DSI (A–C) and colon (D–F) were stained with Rabbit anti-GFP antibodies followed by Anti-Rabbit Cy5 secondary antibodies. Images were captured in the FITC channel (A,D; green) to document endogenous GFP fluorescence, followed by capturing the same region in the Cy5 channel (B,E; red) to document the GFP antibody-stained tissue. These images were overlayed (C,F; yellow). Since the FITC and Cy5 channels are spectrally distinct, this demonstrates the Rabbit anti-GFP antibody is accurately representing endogenous GFP expression in the mouse intestinal blood cell compartment and epithelium. Arrowheads indicate examples of GFP-positive epithelium. (G–J) GFP-expressing epithelium (green) can be identified by co-staining with the epithelial cell marker E-cadherin (red; arrowheads mark yellow co-stained cells in the crypt; arrow marks co-stained cells on the cuff). (I) Higher magnification of white boxed region in panels G & H. (J) Further magnification of red boxed region from panel I. GFP-positive laminia propria can be observed next to a GFP-positive epithelial cell co-staining for E-cadherin. (K–O) The GFP-positive epithelium can be distinguished from the GFP-positive blood cells. Five micron DSI sections were co-stained for GFP and CD45. (K) Most CD45-positive cells reside within the lamina propria of the villus core, however, some lie along the base of the epithelial cells (arrowheads), known as intra-epithelial lymphocytes (IELs). (L) GFP-expressing epithelium (red arrowheads) can be distinguished from GFP-expressing blood cells (white arrowheads) in this co-stained image. (M–O) GFP-expressing epithelium can be distinguished from CD45-positive IELs because while the nuclei of the IEL are small and sit at the base of the epithelial layer (arrowheads), the epithelial cells have larger/longer nuclei oriented in a single layer and the cell extends much further towards the lumen. The epithelial cells that are GFP-positive can be appreciated (brackets) when juxtaposed to GFP-negative epithelial regions and are distinct from IELs (arrowheads). The long columnar shape of the GFP-positive epithelial cells is distinct from blood cells residing in the lamina propria or IELs. Dashed white lines indicate epithelial/mesenchymal border. Bars = 25 µm.
(4.55 MB TIF)

Figure S2 Fluorescence background controls. (A–D) Detection of endogenous GFP fluorescence from a Y01 GFP mouse DSI (A–B) and colon (C–D). WT C57B6 mouse DSI (E–F) and colon (G–H) do not exhibit appreciable levels of autofluorescence in the FITC channel. (I–L) There is no detectable signal in the FITC channel from DSI (I–J) or colon (K–L) that has been stained with Anti-Rabbit Alexa 488 secondary antibody alone. Images in panels B & C were captured under the same configuration, but at one-third the exposure time as panels E,G,I,K. Nuclei are stained with Hoechst dye (A,C,E,G,I,K; blue). Bars = 25 µm.
(1.92 MB TIF)

Figure S3 FISH controls. (A) Five micron cross-section of distal small intestine from a male GFP mouse was stained for GFP and developed with DAB (brown). Both GFP-positive (upper black box) and GFP-negative (lower black box) regions are identified due to the variegation of the GFP expression in this mouse. Y-chromosome can be detected in the nuclei in both boxed regions as magnified in B & C, demonstrating that the Y-chromosome probe can successfully detect Y-chromosome when the GFP antibody and detection reagents are present. (D) A crypt from a female mouse stained with the Y-chromosome probe demonstrating the lack of staining and the specificity of the probe. Nuclei are stained with Hoechst dye. Bars = 25 µm.
(3.26 MB TIF)

Figure S4 Defining Fusion. (A–C) Rosa mice transplanted with GFP-expressing bone marrow were analyzed for fusion in the epithelium by confocal microscopy. Tissue from the distal small intestine were co-stained for GFP (green; donor), β-gal, (red; recipient), laminin (grayscale; laminia propria compartment), and Hoechst (blue; nuclei). Fusion can be detected in the epithelium (brackets) by the co-expression of GFP and β-gal. Dashed white lines indicate epithelial/mesenchymal border and were drawn based on laminin staining from panel A. (D) A broad view of a typical stretch of DSI tissue after transplantation. The GFP-expressing epithelium indicative of fusion is apparent (asterisks). Bars = 25 µm.
(4.64 MB TIF)

Figure S5 Amounts of fusion can be modulated. (A) Wild-type (WT) mice were transplanted with GFP-expressing whole bone marrow (WBM). An example of a stretch of tissue from the colon stained with antibodies against GFP and laminin demonstrated an appreciable amount of GFP-positive fused epithelium (asterisks). (B) When IL-10-/- mice were transplanted with GFP-expressing WBM, the amount of GFP-positive epithelial fusion that could be quantified increased compared to WT (asterisks), but was decreased again when the anti-inflammatory 5-ASA was given (C). Left-hand panels are higher magnifications of boxed regions from the larger stretches of tissue on the right, demonstrating GFP-positive epithelium on the colonic cuffs and in the crypts. Dashed white lines indicate epithelial/mesenchymal border. Bars = 25 µm.
(4.93 MB TIF)

Figure S6 Dextran Sodium Sulfate (DSS) Model of colonic inflammation. After separation, a subset of parabiotic mice were administered dextran sodium sulfate (DSS), to induce inflammation. Wholemount colon from a DSS-treated mouse (B) has major inflammatory changes compared to a wild type mouse (A). This is further appreciated by H&E, where the DSS-treated animal has extensive immune infiltrate (D, arrowheads) compared to the WT colon cross-section (C). Bars in A & B = 1.5 mm. Bars in C & D = 25 µm.
(4.05 MB TIF)

Figure S7 Liver hepatocytes and skeletal muscle have a proliferative response to lethal irradiation. To examine proliferation in the liver and skeletal muscle after irradiation-induced injury, wild type (WT) mice were administered a single lethal dose of whole-body gamma-IR (9Gy). Liver and skeletal muscle (quadricep, tibialis anterior and soleus muscles) were isolated 7 days post-irradiation. (A–D) Liver and skeletal muscle sections from lethally irradiated mice were stained with the proliferative marker Ki67 (green), either FAH (liver; red) or myosin (muscle; red) and Hoechst dye (blue). White arrowheads indicate Ki67-positive nuclei. Boxed regions in A & B are magnified in C & D. (G,H) Quantification of percentage Ki67-positive nuclei revealed that lethally irradiated mice harbored a marked proliferative response in the liver and skeletal muscle when compared to unirradiated control animals.
(2.64 MB TIF)

Table S1 Inflammatory Status. qRT-PCR was carried out for Interleukin-1β (IL-1β and Melanin-concentrating hormone receptor 1 (Mchr1) on various experimental samples to determine changes in inflammatory status. These genes have been demonstrated to increase in an intestinal inflammatory setting in both human and mouse samples. mRNA was isolated from either whole intestine, mesenchyme, or epithelium and cDNA transcribed. Each sample was normalized to Gapdh and compared to its appropriate baseline control. The IL-10-/- samples exhibited decreases when treated with anti-inflammatory drugs, while the AhCre+;Apc-/- proliferative model samples showed no change in inflammatory status when compared to mock-injected controls.
(0.06 MB PDF)
