# Supplementary figures and images for: Inflammation and Proliferation Act Together to Mediate Intestinal Cell Fusion
Source: PLoS One. 2009 Aug 6;4(8):e6530. doi: 10.1371/journal.pone.0006530 (PMC2716548; doi:10.1371/journal.pone.0006530)

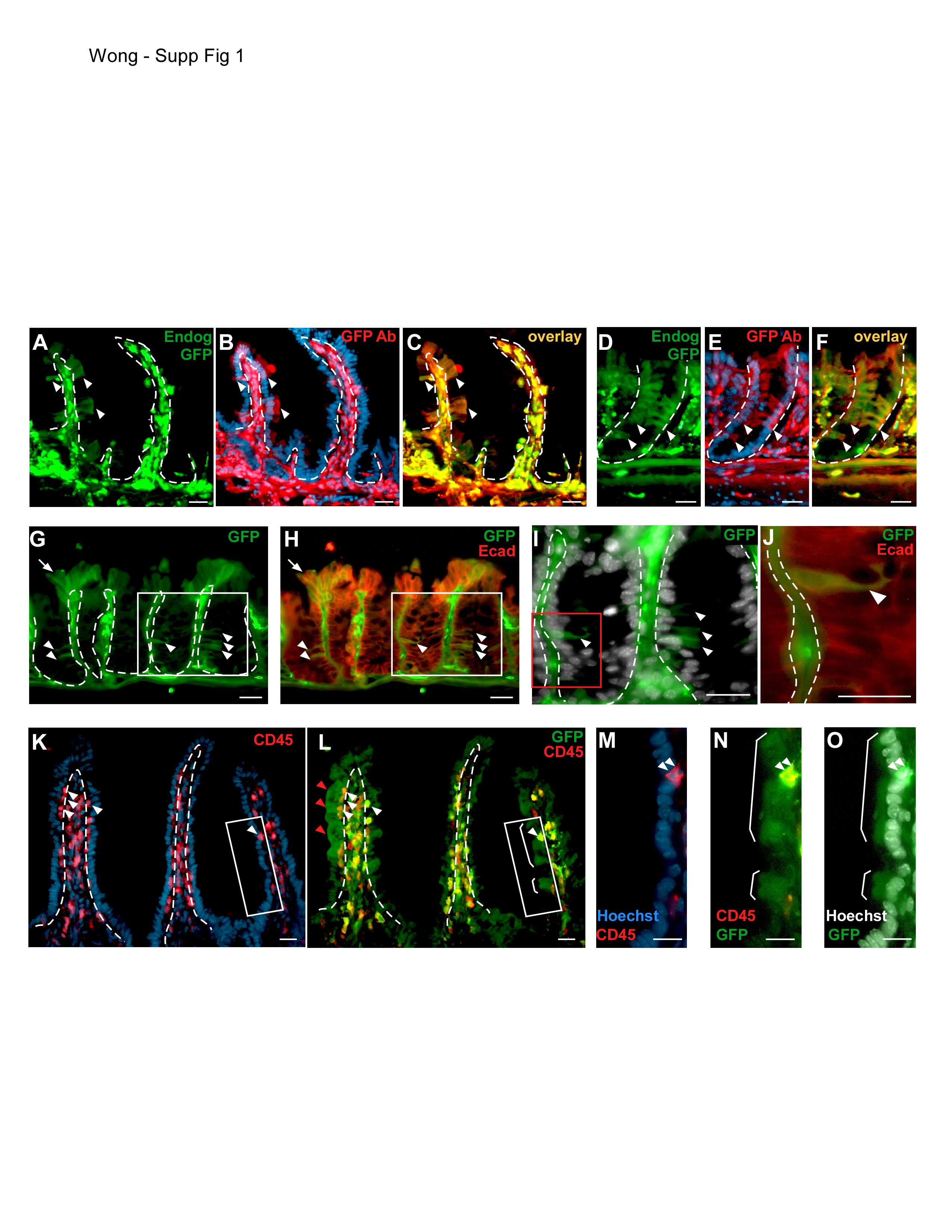

Supplement: Figure S1 — GFP-expressing cell type. Wild-type (WT) mice were transplanted with GFP-expressing whole bone marrow (WBM). (A–F) Five µm sections from DSI (A–C) and colon (D–F) were stained with Rabbit anti-GFP antibodies followed by Anti-Rabbit Cy5 secondary antibodies. Images were captured in the FITC channel (A,D; green) to document endogenous GFP fluorescence, followed by capturing the same region in the Cy5 channel (B,E; red) to document the GFP antibody-stained tissue. These images were overlayed (C,F; yellow). Since the FITC and Cy5 channels are spectrally distinct, this demonstrates the Rabbit anti-GFP antibody is accurately representing endogenous GFP expression in the mouse intestinal blood cell compartment and epithelium. Arrowheads indicate examples of GFP-positive epithelium. (G–J) GFP-expressing epithelium (green) can be identified by co-staining with the epithelial cell marker E-cadherin (red; arrowheads mark yellow co-stained cells in the crypt; arrow marks co-stained cells on the cuff). (I) Higher magnification of white boxed region in panels G & H. (J) Further magnification of red boxed region from panel I. GFP-positive laminia propria can be observed next to a GFP-positive epithelial cell co-staining for E-cadherin. (K–O) The GFP-positive epithelium can be distinguished from the GFP-positive blood cells. Five micron DSI sections were co-stained for GFP and CD45. (K) Most CD45-positive cells reside within the lamina propria of the villus core, however, some lie along the base of the epithelial cells (arrowheads), known as intra-epithelial lymphocytes (IELs). (L) GFP-expressing epithelium (red arrowheads) can be distinguished from GFP-expressing blood cells (white arrowheads) in this co-stained image. (M–O) GFP-expressing epithelium can be distinguished from CD45-positive IELs because while the nuclei of the IEL are small and sit at the base of the epithelial layer (arrowheads), the epithelial cells have larger/longer nuclei oriented in a single layer and the cel [file pone.0006530.s001.tif]

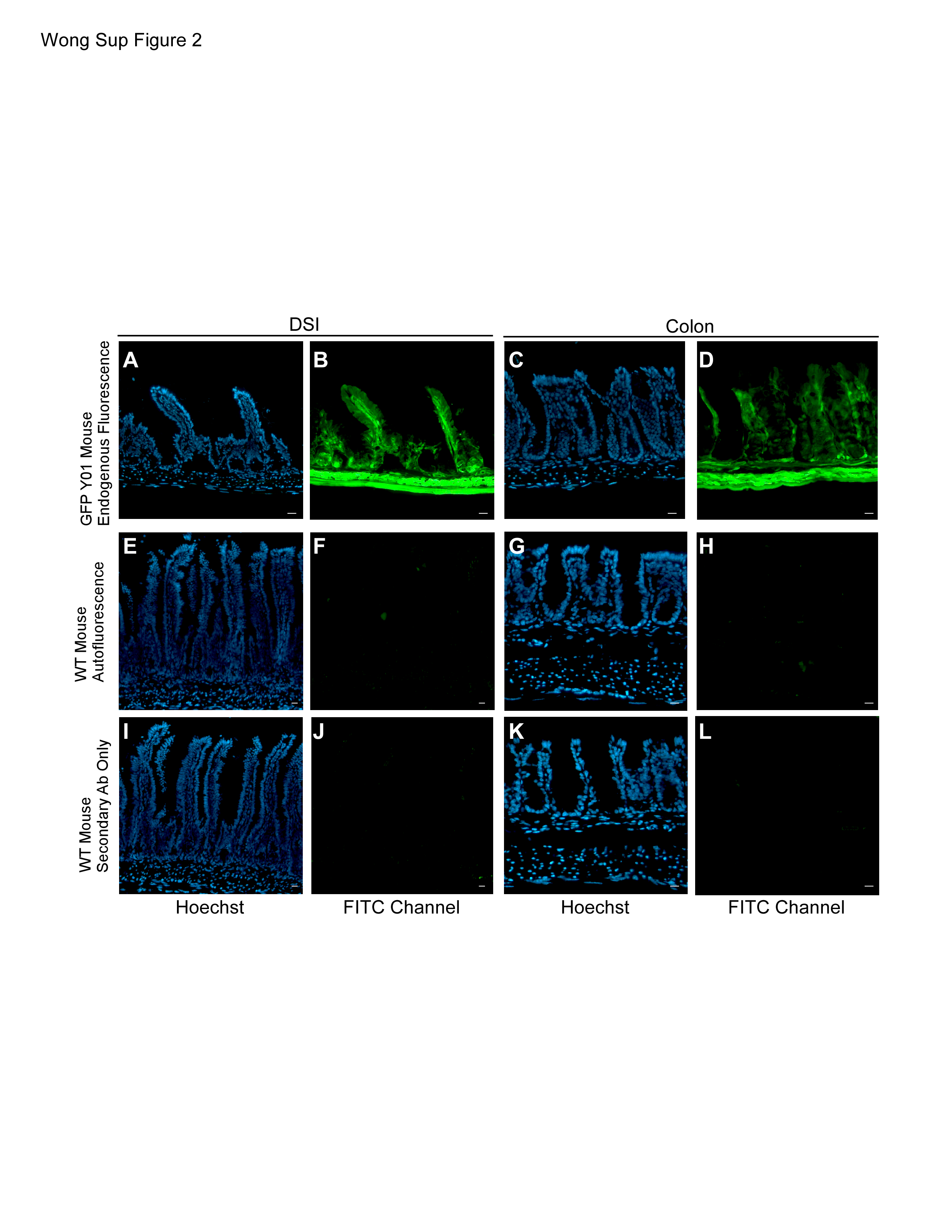

Supplement: Figure S2 — Fluorescence background controls. (A–D) Detection of endogenous GFP fluorescence from a Y01 GFP mouse DSI (A–B) and colon (C–D). WT C57B6 mouse DSI (E–F) and colon (G–H) do not exhibit appreciable levels of autofluorescence in the FITC channel. (I–L) There is no detectable signal in the FITC channel from DSI (I–J) or colon (K–L) that has been stained with Anti-Rabbit Alexa 488 secondary antibody alone. Images in panels B & C were captured under the same configuration, but at one-third the exposure time as panels E,G,I,K. Nuclei are stained with Hoechst dye (A,C,E,G,I,K; blue). Bars = 25 µm. (1.92 MB TIF) [file pone.0006530.s002.tif]

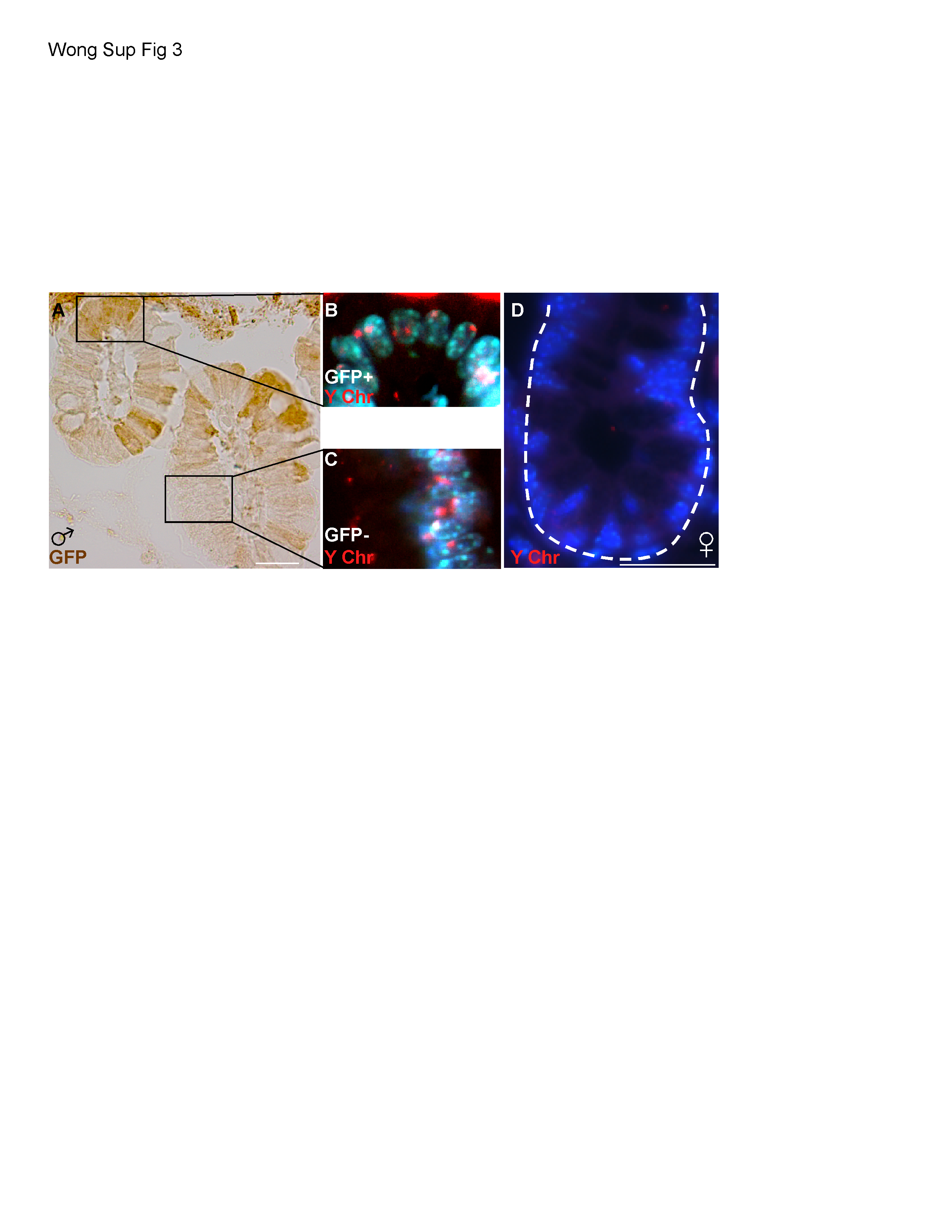

Supplement: Figure S3 — FISH controls. (A) Five micron cross-section of distal small intestine from a male GFP mouse was stained for GFP and developed with DAB (brown). Both GFP-positive (upper black box) and GFP-negative (lower black box) regions are identified due to the variegation of the GFP expression in this mouse. Y-chromosome can be detected in the nuclei in both boxed regions as magnified in B & C, demonstrating that the Y-chromosome probe can successfully detect Y-chromosome when the GFP antibody and detection reagents are present. (D) A crypt from a female mouse stained with the Y-chromosome probe demonstrating the lack of staining and the specificity of the probe. Nuclei are stained with Hoechst dye. Bars = 25 µm. (3.26 MB TIF) [file pone.0006530.s003.tif]

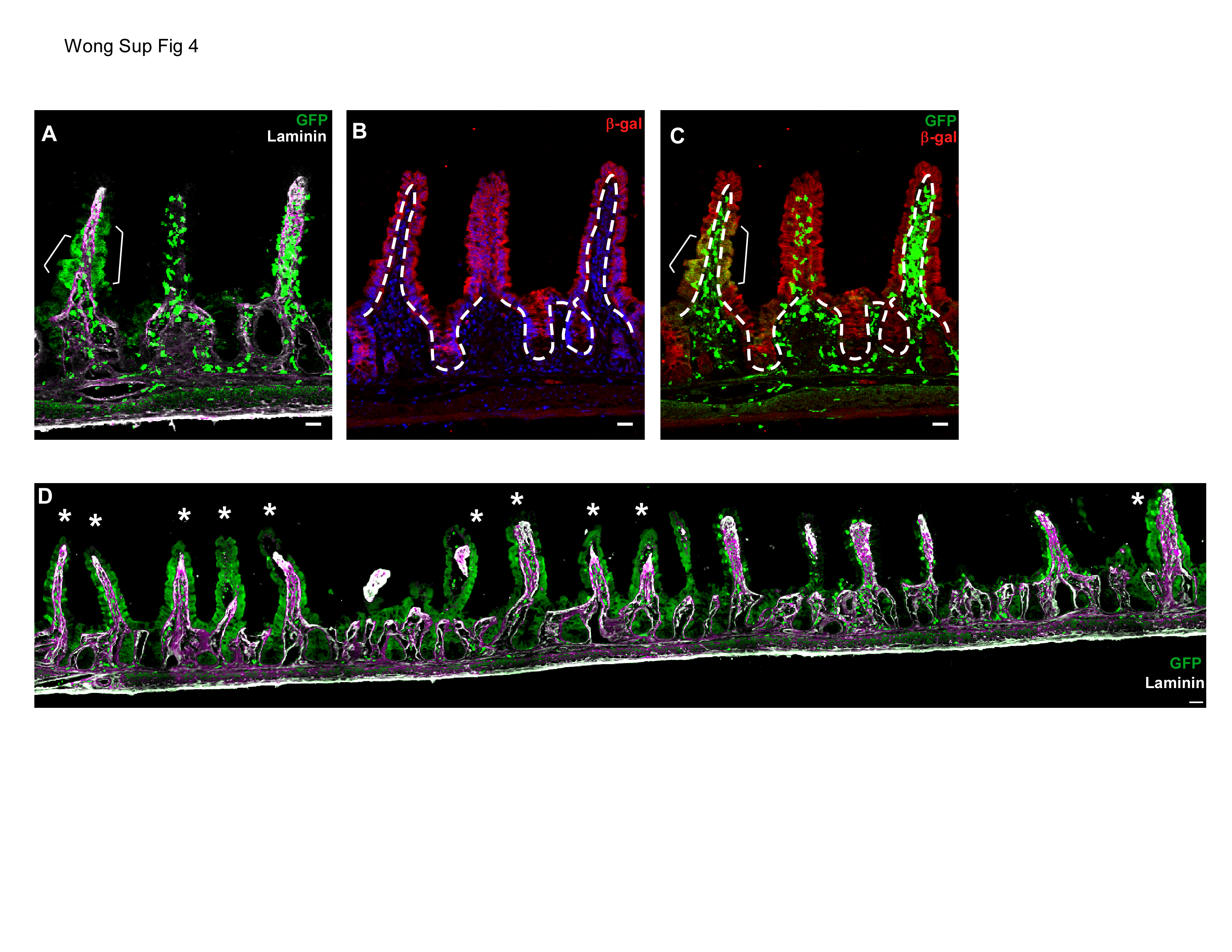

Supplement: Figure S4 — Defining Fusion. (A–C) Rosa mice transplanted with GFP-expressing bone marrow were analyzed for fusion in the epithelium by confocal microscopy. Tissue from the distal small intestine were co-stained for GFP (green; donor), β-gal, (red; recipient), laminin (grayscale; laminia propria compartment), and Hoechst (blue; nuclei). Fusion can be detected in the epithelium (brackets) by the co-expression of GFP and β-gal. Dashed white lines indicate epithelial/mesenchymal border and were drawn based on laminin staining from panel A. (D) A broad view of a typical stretch of DSI tissue after transplantation. The GFP-expressing epithelium indicative of fusion is apparent (asterisks). Bars = 25 µm. (4.64 MB TIF) [file pone.0006530.s004.tif]

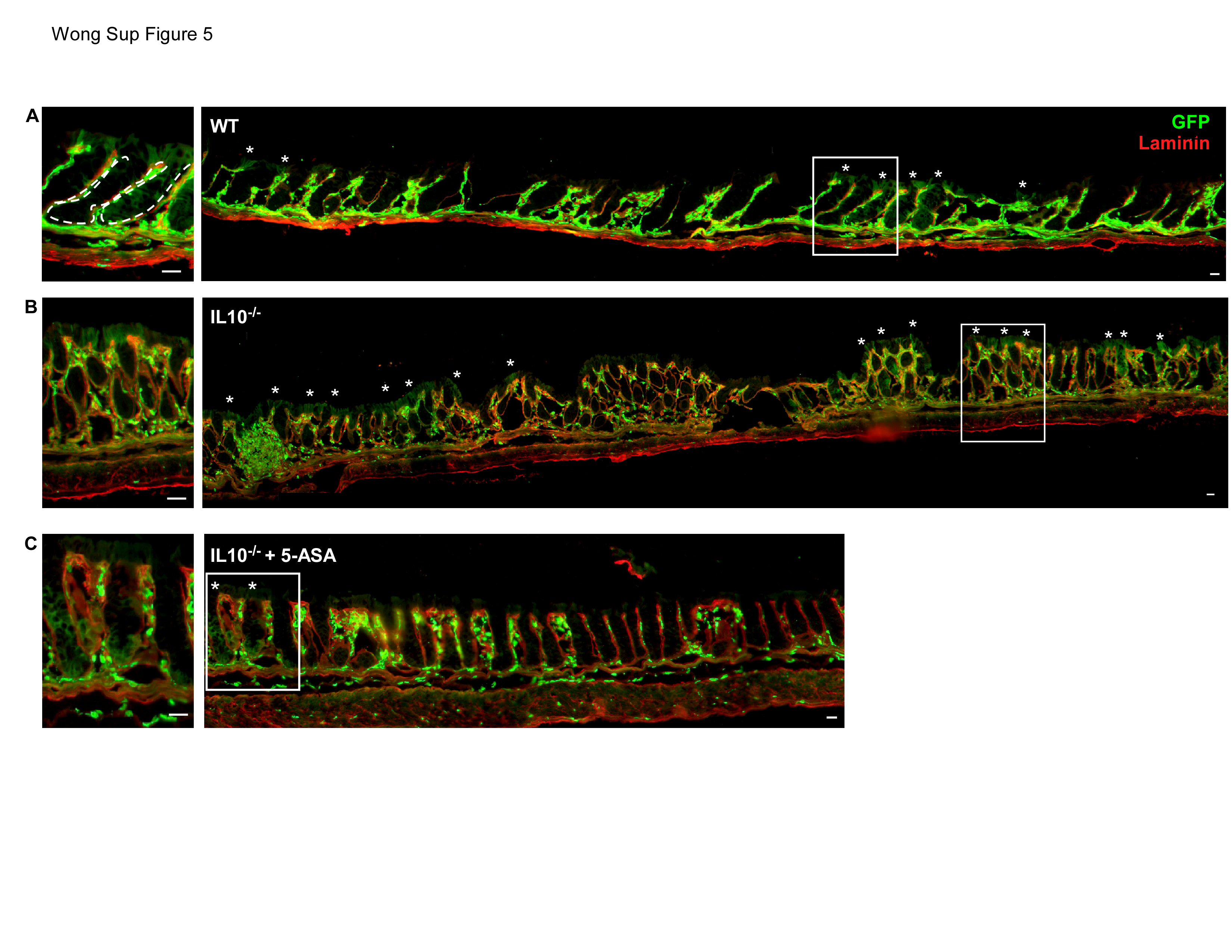

Supplement: Figure S5 — Amounts of fusion can be modulated. (A) Wild-type (WT) mice were transplanted with GFP-expressing whole bone marrow (WBM). An example of a stretch of tissue from the colon stained with antibodies against GFP and laminin demonstrated an appreciable amount of GFP-positive fused epithelium (asterisks). (B) When IL-10-/- mice were transplanted with GFP-expressing WBM, the amount of GFP-positive epithelial fusion that could be quantified increased compared to WT (asterisks), but was decreased again when the anti-inflammatory 5-ASA was given (C). Left-hand panels are higher magnifications of boxed regions from the larger stretches of tissue on the right, demonstrating GFP-positive epithelium on the colonic cuffs and in the crypts. Dashed white lines indicate epithelial/mesenchymal border. Bars = 25 µm. (4.93 MB TIF) [file pone.0006530.s005.tif]

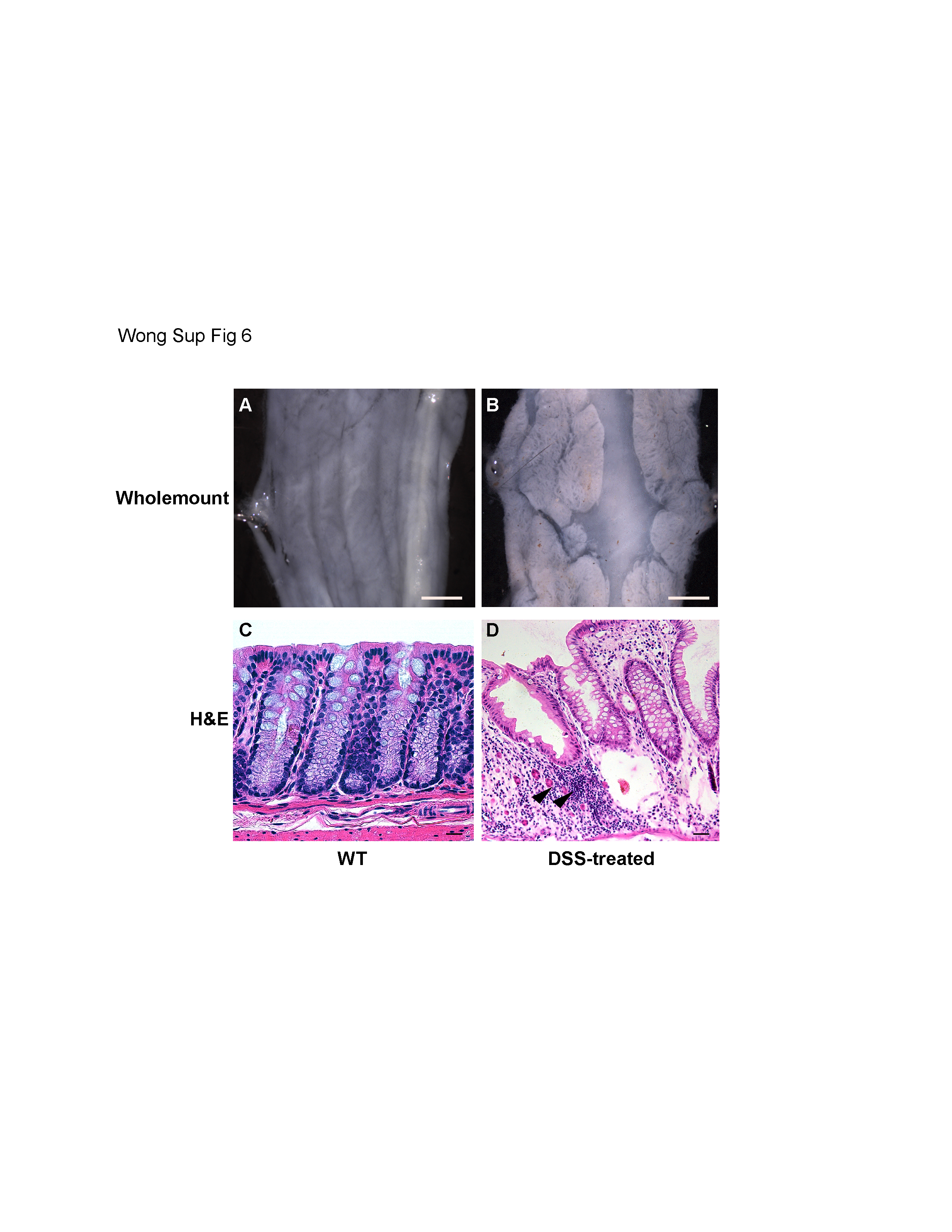

Supplement: Figure S6 — Dextran Sodium Sulfate (DSS) Model of colonic inflammation. After separation, a subset of parabiotic mice were administered dextran sodium sulfate (DSS), to induce inflammation. Wholemount colon from a DSS-treated mouse (B) has major inflammatory changes compared to a wild type mouse (A). This is further appreciated by H&E, where the DSS-treated animal has extensive immune infiltrate (D, arrowheads) compared to the WT colon cross-section (C). Bars in A & B = 1.5 mm. Bars in C & D = 25 µm. (4.05 MB TIF) [file pone.0006530.s006.tif]

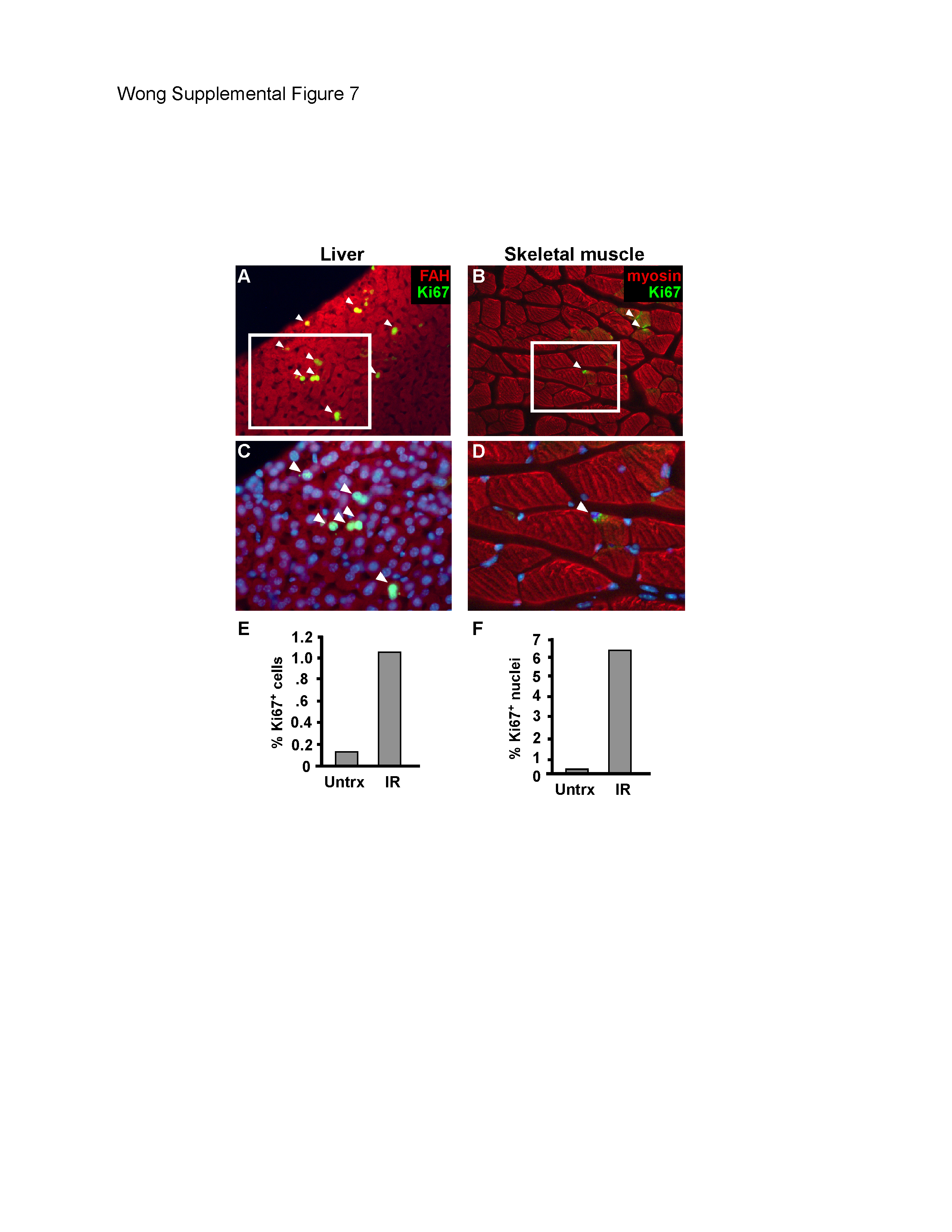

Supplement: Figure S7 — Liver hepatocytes and skeletal muscle have a proliferative response to lethal irradiation. To examine proliferation in the liver and skeletal muscle after irradiation-induced injury, wild type (WT) mice were administered a single lethal dose of whole-body gamma-IR (9Gy). Liver and skeletal muscle (quadricep, tibialis anterior and soleus muscles) were isolated 7 days post-irradiation. (A–D) Liver and skeletal muscle sections from lethally irradiated mice were stained with the proliferative marker Ki67 (green), either FAH (liver; red) or myosin (muscle; red) and Hoechst dye (blue). White arrowheads indicate Ki67-positive nuclei. Boxed regions in A & B are magnified in C & D. (G,H) Quantification of percentage Ki67-positive nuclei revealed that lethally irradiated mice harbored a marked proliferative response in the liver and skeletal muscle when compared to unirradiated control animals. (2.64 MB TIF) [file pone.0006530.s007.tif]
