# Supplementary material for: Inflammation and Proliferation Act Together to Mediate Intestinal Cell Fusion
Source: PLoS One. 2009 Aug 6;4(8):e6530. doi: 10.1371/journal.pone.0006530 (PMC2716548; doi:10.1371/journal.pone.0006530)
Supplement: Table S1 — Inflammatory Status. qRT-PCR was carried out for Interleukin-1β (IL-1β and Melanin-concentrating hormone receptor 1 (Mchr1) on various experimental samples to determine changes in inflammatory status. These genes have been demonstrated to increase in an intestinal inflammatory setting in both human and mouse samples. mRNA was isolated from either whole intestine, mesenchyme, or epithelium and cDNA transcribed. Each sample was normalized to Gapdh and compared to its appropriate baseline control. The IL-10-/- samples exhibited decreases when treated with anti-inflammatory drugs, while the AhCre+;Apc-/- proliferative model samples showed no change in inflammatory status when compared to mock-injected controls. (0.06 MB PDF) [file pone.0006530.s008.pdf]

Supplementary Table-S1 (Wong)

cDNA	Relative to	Fold-Change IL-1β	Fold-Change Mchr1
**$IL10^{-/-}$ Colonic Epithelium**	WT Colonic Epithelium	6.7	N.D.
**$IL10^{-/-}$ Colonic Epithelium + 5-ASA**	$IL10^{-/-}$ Colonic Epithelium	-303	-3.7
**$IL10^{-/-}$ Colonic Mesenchyme + 5-ASA**	$IL10^{-/-}$ Colonic Mesenchyme	-24	N/A[a]
**Induced $AhCre^{+}$;$Apc^{-/-}$ Intestinal Epithelium**	WT Mock Induced Intestinal Epithelium	-1.01	-1.09

[a] not expressed in mesenchyme; N.D., Not Determined
